# Approximate Number Sense in Students With Severe Hearing Loss: A Modality-Neutral Cognitive Ability

**DOI:** 10.3389/fnhum.2021.688144

**Published:** 2021-06-09

**Authors:** Hailin Ma, Xiaoou Bu, Emily M. Sanford, Tongao Zeng, Justin Halberda

**Affiliations:** ^1^College of Education, Shanxi Normal University, Xi’an, China; ^2^Plateau Brain Science Research Center, Tibet University, Lhasa, China; ^3^Faculty of Education, EastChina Normal University, Shanghai, China; ^4^Department of Psychological and Brain Sciences, Johns Hopkins University, Baltimore, MD, United States

**Keywords:** approximate number sense, mathematics, cognition, hearing loss, domain general

## Abstract

The Approximate Number System (ANS) allows humans and non-human animals to estimate large quantities without counting. It is most commonly studied in visual contexts (i.e., with displays containing different numbers of dots), although the ANS may operate on all approximate quantities regardless of modality (e.g., estimating the number of a series of auditory tones). Previous research has shown that there is a link between ANS and mathematics abilities, and that this link is resilient to differences in visual experience (Kanjlia et al., 2018). However, little is known about the function of the ANS and its relationship to mathematics abilities in the absence of other types of sensory input. Here, we investigated the acuity of the ANS and its relationship with mathematics abilities in a group of students from the Sichuan Province in China, half of whom were deaf. We found, consistent with previous research, that ANS acuity improves with age. We found that mathematics ability was predicted by Non-verbal IQ and Inhibitory Control, but not visual working memory capacity or Attention Network efficiencies. Even above and beyond these predictors, ANS ability still accounted for unique variance in mathematics ability. Notably, there was no interaction with hearing, which indicates that the role played by the ANS in explaining mathematics competence is not modulated by hearing capacity. Finally, we found that age, Non-verbal IQ and Visual Working Memory capacity were predictive of ANS performance when controlling for other factors. In fact, although students with hearing loss performed slightly worse than students with normal hearing on the ANS task, hearing was no longer significantly predictive of ANS performance once other factors were taken into account. These results indicate that the ANS is able to develop at a consistent pace with other cognitive abilities in the absence of auditory experience, and that its relationship with mathematics ability is not contingent on sensory input from hearing.

## Introduction

Mathematical competence is essential to a wide range of activities in most modern cultures. Previous studies suggest that math ability predicts a variety of long-term consequences such as job attainment and success (Rivera-Batiz, 1992), socio-economic status (Ritchie and Bates, 2013), and health care decisions (Reyna et al., 2009). A wealth of research suggests that individual differences in math abilities depend on many factors, including home learning environment (LeFevre et al., 2010), teacher characteristics (Klibanoff et al., 2006; Beilock et al., 2010), and domain-general skills such as IQ, working-memory, and inhibitory control (Rohde and Thompson, 2007; Gilmore et al., 2013). Recent evidence suggests that there is also an innate, non-symbolic sense of quantity that gives rise to our basic numerical intuitions. A component of this broader number sense emerges from an evolutionarily and ontogenetically ancient Approximate Number System (ANS) which is present in human infants in the first year of life (Xu and Spelke, 2000; Izard et al., 2009; Feigenson et al., 2013; Starr et al., 2013), as well as exhibited by other non-verbal populations including monkeys, fish, rats, chicks, and birds (Feigenson et al., 2004; Agrillo et al., 2012). The ANS is a mental system of approximate number representations that is activated during symbolic and non-symbolic number tasks, which can be modeled by a series of Gaussian curves organized on a mental number line (Gallistel and Gelman, 1992; Dehaene et al., 2003; Piazza et al., 2004; Mazzocco et al., 2011a,b). The key signature of the ANS is that it represents numerical information in an imprecise way, with the imprecision in its representations increasing with the numerosity. Indexing this signature, and the acuity of the ANS, can be captured by a Weber fraction (*w*) which varies between individuals, where a smaller Weber fraction corresponds to higher precision (Pica et al., 2004; Halberda et al., 2008; Halberda and Feigenson, 2008; Piazza et al., 2010; Libertus et al., 2011; Odic et al., 2014). In accordance with Weber’s Law, the difficulty of discriminating two numerosities depends on their ratio rather than their absolute difference (Piazza et al., 2004). For example, it is equally difficult to distinguish which of 8 vs. 16 is larger as it is to distinguish which of 16 vs. 32 is larger. In humans, ANS acuity increases with age, peaking at around 30 years of age (Halberda et al., 2012).

### Math Achievement and ANS in Students With Hearing Loss (SHL) and Students With Normal Hearing (SNH)

Deaf individuals are generally considered to be lagging behind hearing peers in mathematical tasks across a wide age range (Ansell and Pagliaro, 2006). They show delays in abstract counting and scores on standardized tests (e.g., arithmetical problem solving, logical reasoning, and understanding of fractional concepts) (e.g., in 2–3.5-year-olds, Pagliaro and Kritzer, 2010). Mitchell (2008) found that deaf students are significantly below grade level, exiting high school with about a 5th–6th grade level of mathematical achievement. Previous research has shown that because of the impoverished language environments, their hearing losses and limited access to wide-ranging numerical experience, many Students with Hearing Loss (SHL) are deficient in early quantitative concepts (Nunes, 2004; Kritzer, 2009a; Pagliaro and Kritzer, 2010; Pixner et al., 2014). Madalena et al. (2015) found that SHL with an early exposure to a sign language show better performance than those with a late exposure to the same language. Home environment may differ between typically hearing families and families with a SHL. Indeed, typically hearing families increase the probability of occurrence of informal and natural interactions involving numerical knowledge unconsciously by questioning, asking for clarification, or providing additional information in daily life activities (Kritzer, 2009b; Levine et al., 2010).

The ANS is often assumed to relate to arithmetic performance throughout childhood, adolescence and the adult years and current ANS acuity predicts future math ability (Halberda et al., 2008; Mazzocco et al., 2011b; Libertus et al., 2013). After controlling for scientific ability, writing ability and computer proficiency, the correlation between ANS acuity and mathematical ability of subjects aged 11–85 remained significant across the lifespan (Halberda et al., 2012). In addition, ANS acuity contributes to individual differences not only in the general population, but also in some special groups. Young adults with William’s Syndrome performed poorly on both symbolic math and ANS tasks (Libertus et al., 2014), while Wang et al. (2017) found that ANS acuity was linked to symbolic math performance in gifted adolescents. Others found that students with specific math impairment (dyscalculia) performed significantly more poorly on the ANS task than their typically developing peers; in other words, less precise ANS representations are related to difficulty in mathematics broadly (Geary et al., 2008; Piazza et al., 2010; Bull et al., 2011; Skagerlund and Träff, 2016).

As mentioned above, SHL show a range of mathematical difficulties but whether the mechanism of this difficulty is the same as that of students with normal hearing is not known. For instance, in SHL, it may be that the innate ANS representations are as precise as their peers, while the mapping between ANS and more complicated mathematical concepts is delayed due to reduced access to linguistic and mathematical input. If the differences found between SHL and SNH in mathematics performance are due to their differences in experience rather than a difference in their innate ANS representations, there remains a question of whether it is due to a general lack of auditory input, related to delays in access to language or higher-level math concepts, or due to fundamental differences in information processing among SHL (Bull, 2008). In the present study, we aimed to document the potential relationship between hearing loss (and the many factors that covary with it) and ANS acuity.

### ANS and Domain-General Abilities

Because of the potential importance of domain-general abilities to both formal mathematics success and developing ANS acuity, we considered multiple examples of such abilities in the present study.

### Inhibition

Inhibition is thought to be important to performance in ANS tasks. Performance on trials where spatial characteristics can vary widely, e.g., in stimuli that are congruent or incongruent with numerical information. For instance, Clayton et al. (2015) found that people are much more accurate on trials where the larger set numerically also has the larger convex hull (congruent trials) than on trials where the opposite is the case (incongruent trials). Other non-numerical features that can influence responses on number tasks include surface area, diameter, perimeter, and density (Dakin et al., 2011; Gebuis and Reynvoet, 2011).

In order to reduce the extent to which subjects in ANS experiments can rely on non-numerical cues, one strategy has been to use two interleaved groups of stimuli: one where the average dot size is constant across both sets (such that the more numerous set also has a larger e.g., total dot area), and one where the total dot area is constant across both sets (such that the more numerous set has a smaller average dot size; Halberda et al., 2012). By mixing presentations of trials from these two stimulus sets together, neither average dot size nor total area is a reliable predictor of the number of objects throughout the experiment. If a subject tends to rely on a continuous feature such as dot size, then they will show different performance on size congruent and incongruent trials: this feature will help them respond correctly on congruent trials but will result in worse performance on incongruent trials, unless they are able to selectively suppress that signal on incongruent trials. Therefore, in order to consistently perform well on both congruent and incongruent trials, one must exert inhibitory control not unlike that required for a Stroop task. Relative differences in performance between congruent and incongruent trials may reflect differences in inhibitory control across subjects. Given that SHL are often reported to have inhibitory difficulties (Titus, 1995; Traxler, 2000), it may be that part of the source of SHL’s mathematical challenges come from a relative lack of inhibitory control.

### Visual Working Memory

Visual-spatial processing is important for number perception, possibly because of the important role it plays in the formation of set representations from visual sets (Paul et al., 2017). In fact, visual form perception and visual short-term memory have been found to fully account for the relationship between ANS acuity and arithmetic performance in some instances (Zhang et al., 2019). This may be particularly important in young children, who appear to use visuospatial strategies when performing mental arithmetic more than older children (McKenzie et al., 2003), and where visual-spatial short-term memory span increases from 3 to 8 years of age (Pailian et al., 2016), and where visual-spatial short-term memory span has been found to be selectively predictive of math success in young children (Bull et al., 2008).

Further support that visual-spatial processing and working memory are important for number perception comes in the form of co-occurring challenges with number processing and working memory. Deficits in visual-spatial working memory have been found to be associated with numerical magnitude processing weaknesses in children with mathematical learning disabilities (Andersson and Östergren, 2012). Children with developmental dyscalculia have shown math-specific impairments as well as deficits in visuo-spatial working and short-term memory and inhibitory control (Szucs et al., 2013). Notably, ANS acuity differences between typically developing children and children with developmental dyscalculia have been found to be more extreme on size-incongruent trials than size-congruent trials. Because of the role that visual working memory plays in extracting numerical information from visual scenes, it may be extremely important to investigate in situations where ANS acuity varies between populations (Bugden and Ansari, 2015).

Conflicting evidence suggests that visual working memory capacity cannot fully explain numerical deficits. For instance, Peng et al. (2017) found that numerical knowledge mediates the relationship between ANS performance and early arithmetic abilities, above and beyond that which is explained by visuospatial processing. Additionally, research on children born extremely preterm found ANS acuity deficits that were not explainable on the basis of working memory or attention abilities (Libertus et al., 2017). Further research is necessary to investigate the extent to which visual working memory capacity can explain ANS acuity differences between populations. Given that deaf children have been shown to have deficits in visual working memory (López-Crespo et al., 2012), this question is particularly relevant for the current study.

### Attention Network

Numerical processing involves the deployment of attention, more so for subitizing than for large number processing (Anobile et al., 2012). In fact, some studies have found that estimation of large numbers is relatively unaffected by tasks with conflicting attentional demands (Burr et al., 2010). Nonetheless, spatial attention has sometimes been found to be involved in ANS task performance (Anobile et al., 2012).

When studying attention related to other cognitive abilities, the attentional system is sometimes divided into three separate components: alerting, orienting, and executive control attention networks (Fan et al., 2009). The alerting portion refers to the ability to increase attention at the expected onset of a new stimulus. The orienting attention network is thought to explain the ability to select a particular target for attention among a variety of inputs, whether intentional or through attention capture. Finally, the executive control network is thought to detect and resolve conflicts between co-occurring mental computations.

Attentional network development has been a topic of particular interest in deaf children (Daza and Phillips-Silver, 2013). The development of the alerting network is thought to be impaired in the absence of auditory stimulation, while some components of the orienting attention network are enhanced, such as moving and engaging. The executive control network has been found to develop along a similar trajectory to that of hearing children.

There is known to be a strong relationship between number processing, math ability, and attention (Anobile et al., 2013). Like ANS perception, performance on attention tasks has been found to predict symbolic math achievement in children and was also predictive of ANS ability (Anobile et al., 2013). Attentional deficits may be implicated in math-specific disabilities such as developmental dyscalculia. Therefore, we are interested in whether similar attentional deficits impact the numerical processing of SHL, and whether these deficits can be traced to specific attentional networks.

### Summary: Motivations for the Current Work

Considering the ways in which school mathematics abilities might be related to the ANS, and vice versa, it is likely to be a highly interdependent relationship. The Defective Number Module Hypothesis, perhaps too simply, suggests that mathematical deficits may have their roots in innate difficulties processing non-symbolic number; for instance, an impairment of the ANS has been proposed as the origin of dyscalculia, a mathematics-specific learning disability (Butterworth, 2005; Mazzocco et al., 2011a). Of course, an effect in the opposite direction might also occur. Such a relationship might be explored in SHL; not because SHL necessarily have dyscalculia themselves; but rather, because the reduced exposure of SHL to numerical concepts in early development may lead to similar problems (Swanwick et al., 2005). A relatively small number of studies have investigated the performance of SHL on specific areas of mathematics (Pagliaro and Kritzer, 2010). The current study expands this by focusing both on symbolic mathematics and non-symbolic numerical processing.

In Experiment 1, we tested whether SHL’s responses to the ANS task conform to Weber’s law, and investigated whether their acuity is affected by size congruency manipulations (e.g., Clayton et al., 2015). We expected that SHL’s ANS responses will follow Weber’s law, and that they will perform better on size-congruent than size-incongruent trials – just as SNH.

In Experiment 2, we compared SHL to a population of SNH, to test whether effects such as size congruency influence ANS acuity similarly between the two groups. It is possible that congruency manipulations would be especially detrimental to SHL, since they may have particular difficulties with inhibition (Titus, 1995; Traxler, 2000), and inhibition ability is thought to play an important role in mitigating the influence of size congruency on number responses (Clayton and Gilmore, 2015; Norris and Castronovo, 2016). We then explored the extent to which ANS acuity predicts mathematics ability when taking into account other factors such as inhibitory control, visual working memory capacity, and attention network performance. If SHL perform like other students their age, we would expect to see Weber fraction uniquely account for mathematics ability, above and beyond the contributions of these other factors (Halberda et al., 2008; Chen and Li, 2014; Schneider et al., 2016).

## Experiment 1

### Materials and Methods

#### Participants

One hundred and forty-four students with hearing loss (mean age = 13.58 years, *SD* = 2.34, range = 8–18 years; 60 females) from 6 special education schools participated in the study. All were enrolled in the third grade to ninth grade. SHL were prelingually deaf students and exhibited severe (71–90 dB) and profound hearing loss (>91 dB). All of them were right-handed, with normal or corrected-to-normal vision and no history of neurological or psychiatric illness.

#### ANS Acuity

We administered a version of Panamath (Psychophysical Assessment of Number-Sense Acuity; www.panamath.org), a non-symbolic numerical comparison task, to assess the acuity of children’s ANS. The two spatially intermixed arrays of blue and yellow dots were presented for 1,200 ms followed by a 200 ms backward mask, followed by a blank gray screen until the response was completed. Students were asked to judge whether more of the dots were blue or yellow. There were between 5 and 21 dots in each array, the ratios were categorized into 4 ratio bins: 1.14, 1.2, 1.33, and 2, with 20 trials in each ratio bin, yielding a total of 80 trials. To avoid subjects from relying on the cumulative area of dots, on half of the trials dots were size-confounded, and on the other half of the trials dots were size-controlled. Notice that Panamath does not systematically control for all possible non-numerical cues (e.g., convex hull is only partially controlled via the total area and dot size manipulations). Our aims were to test for Weber’s law and (in Experiment 2) to test for the relationship of ANS acuity to formal math abilities. Our interest in inhibitory control was test here only by our area manipulation.

### Results and Discussion

**FIGURE 1:**
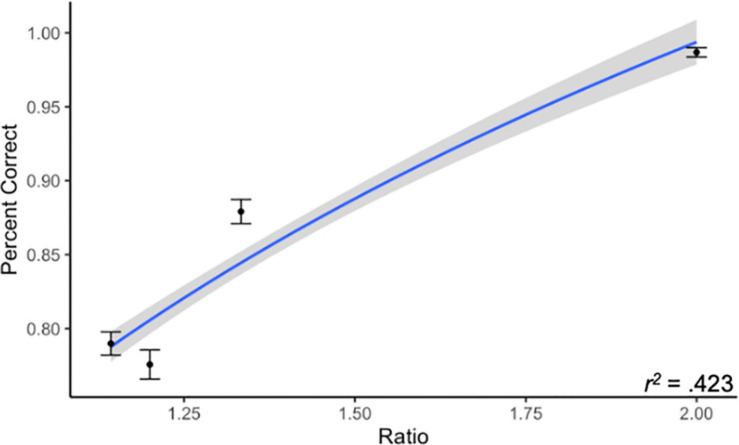
Accuracy by trial ratio. We found that subjects in Experiment 1 conformed to Weber’s law, where accuracy increases as a function of ratio. Line represents best-fitting logarithmic relationship between ratio and percent correct. Gray region represents 95% CI.

Overall, subjects in Experiment 1 had relatively high accuracy on the ANS task (*M* = 85.8%, *SD* = 6.2%). We confirmed that accuracy improved as a logarithmic function of increasing ratio, as is expected with data conforming to Weber’s law (Dehaene, 2003). We evaluated this by performing a linear regression predicting subjects’ average accuracy (on both trial types) from the logarithm of trial ratio. We found that this model significantly predicted accuracy, β = 0.651, *t*(574) = 20.55, *p* < 0.001 (see Figure 1). This result indicates that, among these subjects, accuracy was dependent upon the trial difficulty as determined by comparison ratio, consistent with Weber’s law.

Next, we were interested in whether ANS performance improved with age. We were also interested in whether performance was better on size-congruent trials than size-incongruent trials. Both of these effects have been found repeatedly in previous research on the ANS (e.g., Halberda et al., 2008, 2012; Clayton et al., 2015; Smets et al., 2015).

We used subjects’ accuracy (for all trials, as well as separately for size-congruent and size-incongruent trials) to different ratios (*r*) to fit their Weber fraction (*w*) according to the following psychophysical model, used extensively in previous ANS research (Pica et al., 2004; Cantlon and Brannon, 2006; Halberda and Feigenson, 2008; Halberda et al., 2008, 2012; Piazza et al., 2010; Libertus et al., 2011, 2013, 2014; Odic et al., 2013, 2014; DeWind et al., 2015; DeWind and Brannon, 2016; Starr et al., 2017; Wang et al., 2017):

$$p\,r\,o\,b\,a\,b\,i\,l\,i\,t\,y\,c\,o\,r\,r\,e\,c\,t=1-\frac{1}{2}\,e\,r\,f\,c\,\left(\frac{r-1}{w\,\sqrt{2}\,\sqrt{1+r^{2}}}\right)$$

The model was fit to each subjects’ data using Maximum Likelihood Estimation (MLE) in R. Previous research has indicated that accuracy and response time may index different abilities (e.g., Halberda et al., 2012), and because we were interested in the amount of internal noise in our subjects’ number representations, we focused on using accuracy-based Weber fractions to test our hypotheses.

In this model, a smaller Weber fraction corresponds to higher accuracy and therefore better performance. On average, the subjects in this study had a mean Weber fraction of 0.168, which is in line with previous research on ANS acuity among 14-year-olds (the mean age of our participants), who have been found to have Weber fractions ranging from 0.119 to 0.567 (Halberda et al., 2008).

To evaluate whether performance improved with age, we performed a linear regression predicting Weber fraction (based on all trials) from subject age, expecting to see a negative linear trend (indicating that performance improved with age). Indeed, that was what we found: increasing age significantly predicted a decline in Weber fraction, β = −0.212, *F*(1, 142) = 6.69, *p* = 0.011, *R*^2^ = 0.04.

Next, we investigated whether subjects performed differently on the size-confounded versus size-controlled trials, expecting that subjects would have higher Weber fractions (i.e., worse performance) on size-controlled trials than size-confounded trials. A paired t-test confirmed that subjects had smaller Weber fractions and therefore performed better on the size-confounded (*M* = 0.15, *SD* = 0.09) than size-controlled (*M* = 0.19, *SD* = 0.12) trials, *t*(143) = 4.11, *p* < 0.001.

This preliminary study demonstrates our ability to work with SHL in the relevant schools, and replicates several key findings from the literature on the ANS.

## Experiment 2

### Materials and Methods

#### Participants

In Experiment 2, we focused on a subgroup of the children from Experiment 1 and also ran a new group of age-relevant controls. In order to focus on effects related to symbolic math development, we relied on the Chinese Rating Scale of Pupil’s Mathematic Abilities (C-RSPMA; Wu and Li, 2005) which is normed for children in primary school. For this reason, we restricted our SHL sample to children in primary school with complete datasets as well as a new group of control children with complete data sets. Ninety-seven SHL (*M*_age_ = 12.58 years, *SD* = 1.95, range = 8–18 years; 38 females) from 6 special education schools and 97 SNH (*M*_age_ = 10.36 years, *SD* = 1.24, range = 8–12 years; 47 females) from 1 normal primary school in Sichuan, China, participated in the study. All were enrolled in the third grade to sixth grade. The SNH students were approximately matched to the SHL in grade level (although SHL were on average older than SNH and had a much wider age range, as is typical in SHL). SHL were prelingually deaf students and exhibited severe (71–90 dB) and profound hearing loss (>91 dB). All subjects were right-handed, with normal or corrected-to-normal vision and no history of neurological or psychiatric illness. Table 1 shows detailed demographic information on all participants.

**TABLE 1:** Experiment 2 participant demographic information.

	**SHL**			**SNH**		
	**Age (M ± SD)**	**Sex**		**Age(M ± SD)**	**Sex**	
**Group**		**Male**	**Female**		**Male**	**Female**
3 grade	11.57 ± 1.93	14	9	8.70 ± .56	10	13
4 grade	12.35 ± 1.90	15	8	9.84 ± .36	11	11
5 grade	12.14 ± .94	12	10	10.83 ± .39	12	11
6 grade	13.93 ± 1.93	18	11	11.72 ± .45	17	12

#### Tasks and Procedure

##### Chinese rating scale of pupil’s mathematic abilities

The Chinese Rating Scale of Pupil’s Mathematic Abilities (C-RSPMA; Wu and Li, 2005) based on the Germany Rating Scale of Pupil’s Mathematic Abilities established by Heidelberg University was used to assess the primary students’ basic mathematical competencies.

C-RSPMA is composed of 11 subtests divided into two broad categories. One category tests mathematics operation such as addition, subtraction, multiplication, division, blank filling and comparisons. The other category focuses on skills in spatial vision and logical thinking, with tasks such as figure writing, length estimation, block counting, graph counting and figure connection. For these 11 subtests, students were required to answer as many items as possible within the stipulated time (1–3 min, dependent on different subtests). The Cronbach’s alpha is above 0.7, split-half reliability coefficient is 0.83.

##### Non-verbal IQ

To evaluate children’s non-verbal IQ, we administered the combined Raven’s Test (CRT-CC3; Wang et al., 2007). This test contains 72 matrices of increasing difficulty, and a correct answer yielded one point. Students were required to identify the missing element that best completes a pattern from six or eight alternatives.

##### Inhibition

**FIGURE 2:**
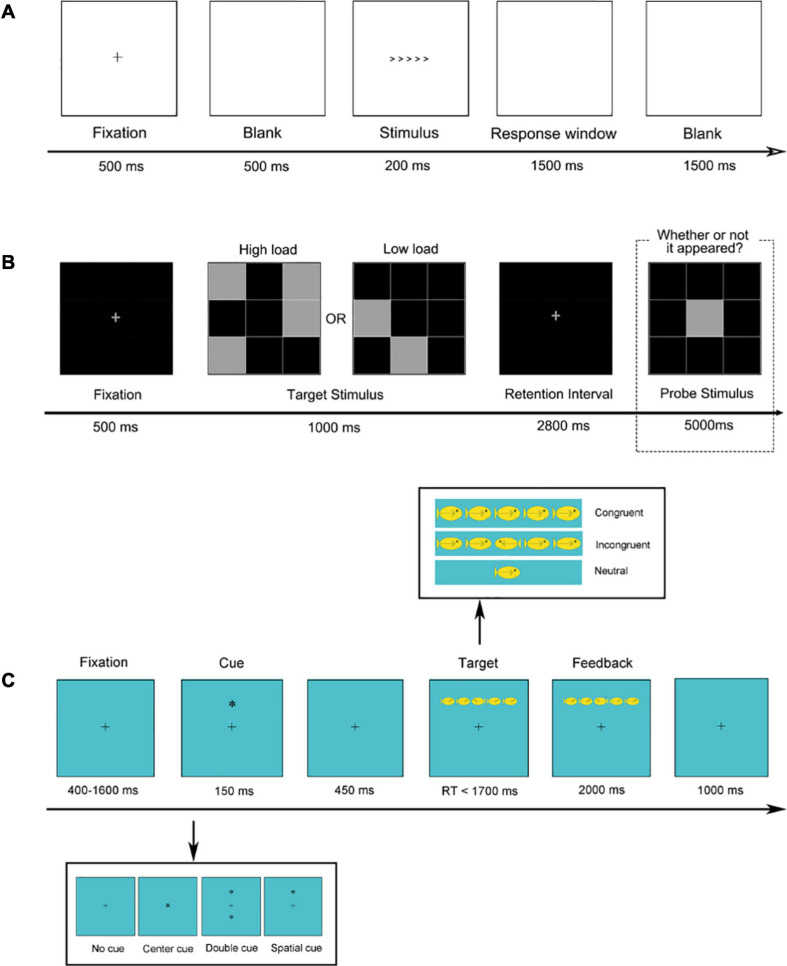
The flowchart of the experimental paradigms. **(A)** The flanker task. **(B)** Visual delayed match-to-sample task. **(C)** Attention network task.

The Flanker Task was used to measure inhibitory control (Eriksen and Eriksen, 1974; see Figure 2A). This task measures inhibitory control by requiring subjects to respond in the direction of a central arrow while ignoring the sometimes-conflicting direction of the arrows on either side of it. Each trial started with a fixation cross presented centrally for 500 ms followed by a blank screen for 500 ms, after which the target and flanking stimuli appeared. These stimuli were presented for 200 ms followed by a response window until a response was made up to 1,500 ms later. A blank screen of 1,500 ms separated each trial. Half of the trials were congruent (<<<<< or >>>>>), whereas the other half were incongruent (e.g., <<><< or >><>>). Students were instructed to respond as accurately and as quickly as possible to indicate the direction of the centrally presented target arrow by key press. This task contained a practice block with 12 trials and two experimental blocks with 60 trials each.

##### Visual working memory

We used a visual delayed match-to-sample task to measure visual working memory (Dong et al., 2014; Figure 2B). A fixation cross was presented for 500 ms followed by a target stimulus, which was a grid that had some squares highlighted (high load condition: 4/9 highlighted; low load condition: 2/9 highlighted). The target stimulus was visible for 1,000 ms, followed by a blank screen for 2,800 ms (where the participant had to hold the locations of the target squares in memory). Finally, a probe stimulus appeared, which consisted of the same grid but with only one square highlighted. Students indicated by button press whether or not the probe square appeared in one of the same locations as was highlighted in the target stimulus. The probe stimulus was visible for up to 5,000 ms. This task contained a practice block with 10 trials and one experimental block with 60 trials.

##### Attention network

The attention network test is used to measure the efficiency of the three aspects of attentional networks (i.e., alerting, orienting, and conflict; Fan et al., 2002; Rueda et al., 2004; Figure 2C). Each trial began with a fixation presented at the center of the screen for a random duration between 400 and 1,600 ms, after which the cue stimulus appeared for 150 ms. Subsequently, the fixation was again presented for 450 ms followed by a target stimulus which appeared for a maximum duration of 1,700 ms, followed by feedback for 2,000 ms. Finally, a fixation of 1,000 ms separated each trial. This task consisted of one practice block of 12 trials and two experimental blocks involving 60 trials each.

The ANT includes four cue conditions (no cue, central cue, double cue, and spatial cue) and three target conditions (congruent, incongruent, and neutral). The target stimulus was a single yellow fish or a horizontal row of five yellow fish which were presented about 1∘either above or below fixation. Each fish subtended 0.58∘ of visual angle and was separated from neighboring fish by 0.21∘. The five fish subtended a total of 8.84∘. Students were instructed to respond to the direction that a central fish was facing by button press.

##### ANS acuity

The procedure to evaluate ANS acuity was the same as Experiment 1.

##### Data preparation

The data preparation and Weber fraction modeling for the ANS results were identical to those used in Experiment 1.

The C-RSPMA was scored following standard protocol to calculate a Mathematics score for each subject (Wu and Li, 2005).

For the task measuring Non-verbal IQ, the final raw Raven test scores were converted to a standard IQ score according to the norm for Chinese children.

For the task measuring Inhibitory Control, we computed a score based on response time in the Flanker Task. An index of inhibitory control for each subject was calculated using the following formula over mean response times in the two conditions: Score = RT_incongruent_ – RT_congruent_. This single value represents how much longer it took the subject to respond to incongruent trials than to congruent trials, and therefore a lower value corresponds to better inhibitory control.

For the visual working memory task, a composite score was created for working memory performance by combining results from both accuracy and response time. Across all subjects, we z-scored average response times on high memory load trials (correct responses only), average response times on low memory load trials (correct responses only), average accuracy on high memory load trials, and average accuracy on low memory load trials. This resulted in each subject having four values that indicated how well, relative to other subjects, they performed on each of these four indices of performance. We then averaged these four z-scores for each subject to get a single composite score of performance on the working memory task relative to other subjects in the sample.

For the Attention Network Task, we calculated a separate score for the efficiency of the three attentional networks based on response times to different cue conditions. The efficiency of three attentional network scores based on the RTs were calculated using the following formula (see Figure 2C for cue conditions): Alerting effect = RT_no__–__cue_ − RT_double__–__cue_, Orienting effect = RT_center__–__cue_ − RT_spatial__–__cue_, and Conflict effect = RT_incongruent_ − RT_congruent_.

### Results

#### ANS Performance

**FIGURE 3:**
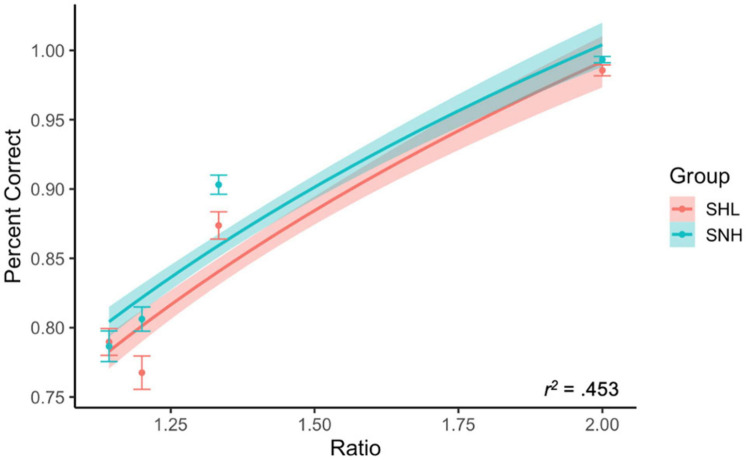
Accuracy by trial ratio and group membership. As expected by Weber’s law, accuracy increased as a function of trial ratio. SNH were, on average, more accurate than SHL. There was no interaction between these two variables. Lines represents best-fitting logarithmic relationship between ratio and percent correct. Shading corresponds to 95% CI.

Once again, our subjects performed fairly well in terms of accuracy on the ANS task (*M* = 86.3%, *SD* = 5.6%). We once again evaluated whether accuracy was dependent upon trial ratio to confirm that our results were consistent with Weber’s law. We used multiple regression predicting accuracy from the logarithm of trial ratio, group membership (SNH or SHL), and their interaction. We expected that both groups would show a significant influence of trial ratio on accuracy, and that there would be no interaction between the two variables. This was confirmed: the logarithm of trial ratio significantly predicted accuracy, β = 0.685, *t*(772) = 18.25, *p* < 0.001. There was also a significant effect of group membership, where SNH (*M* = 87.2%, *SD* = 4.6%) had slightly higher accuracy on average than SHL (*M* = 85.4%, *SD* = 6.3%), β = 0.076, *t*(772) = 2.85, *p* = 0.004. There was no interaction between the two, indicating that trial difficulty impacted both groups the same relative amount, *p* = 0.579 (see Figure 3).

Next, we were interested in performance as indexed by model-fitted Weber fractions. Overall, with one Weber fraction fit to each subject’s responses to all trials, our subjects had similar Weber fractions to those we found in Experiment 1 (*M* = 0.162, *SD* = 0.065).

**FIGURE 4:**
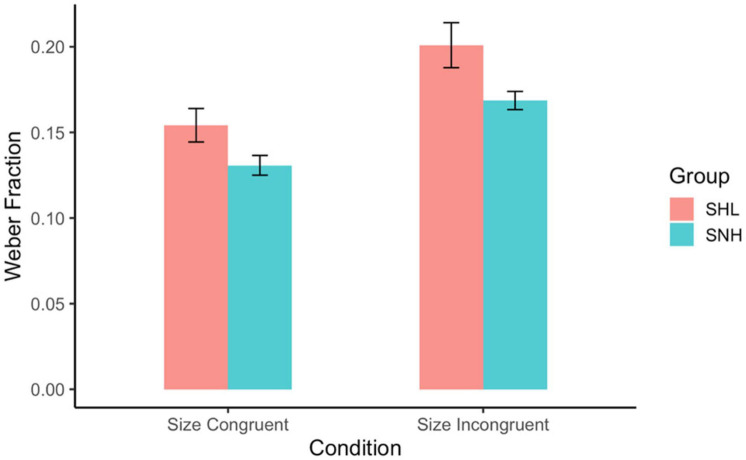
Weber fractions by group membership and size-congruity. Weber fractions were lower (corresponding to more precise responses) when subjects were responding to size-congruent trials than when they were responding to size-incongruent trials. Also, SNH had higher acuity than SHL. There was no interaction between these two variables. Error bars represent standard error.

We were interested in whether Weber fraction was affected by group membership (SHL vs. SNH) and size congruity (congruent vs. incongruent trials). To test this, we again fit each subject’s responses with a Weber fraction, separately for size congruent and incongruent trials. Then we conducted a two-way ANOVA predicting Weber fraction from hearing and size congruency, with group membership as a between-subjects variable and size congruency as a within-subjects variable. Both main effects were significant. Consistent with the results from Experiment 1, we found that acuity was better on size-congruent trials (*M* = 0.142, *SD* = 0.080) than on size-incongruent trials (*M* = 0.185, *SD* = 0.100), *F*(1, 382) = 5.044, *p* = 0.025, across the two groups. For group membership, we found that SHL (*M* = 0.174, *SD* = 0.079) had larger Weber fractions than SNH (*M* = 0.150, *SD* = 0.043), *F*(1, 382) = 7.34, *p* = 0.007, indicating that SNH had slightly better acuity. Importantly, there was no significant interaction between these two factors, *F*(1, 382) = 0.29, *p* = 0.634 (see Figure 4). This indicates that size-congruency impacted performance equally for subjects regardless of group membership—which runs counter to the expectation that difficulty with inhibition would drive especially worse performance for SHL on size-incongruent trials.

**FIGURE 5:**
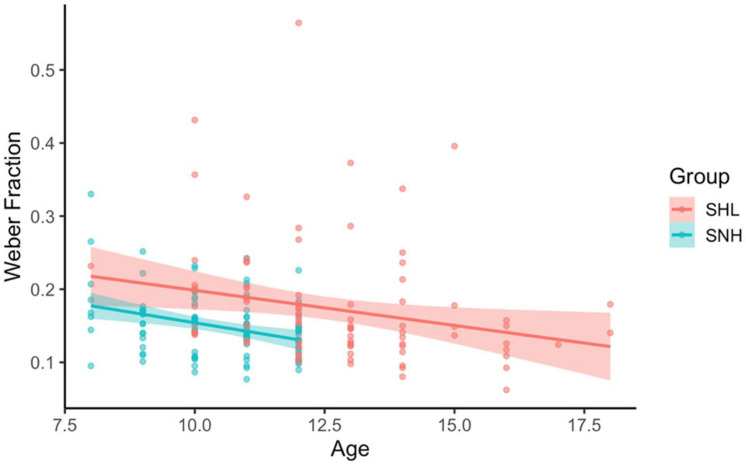
Weber fraction by age and group membership. In both groups, increasing age was linked with decreasing Weber fractions. Line corresponds to best-fitting linear relationship between Age and Weber fraction for each group. Shading corresponds to 95% CI.

We also verified whether Weber fraction varied with age in this sample. We investigated this by performing a linear regression predicting Weber fraction (collapsed across congruency conditions) from age and group membership. Consistent with previous research, we found that the model significantly predicted Weber fractions, *F*(3, 190) = 7.03, *p* < 0.001, *R*^2^ = 0.09 (see Figure 5). Both group membership, β = −0.366, *t*(190) = 4.24, *p* < 0.001, and age, β = −0.293, *t*(190) = 2.96, *p* = 0.003, significantly predicted Weber fractions, while their interaction was not significant, *p* = 0.741. Within both groups, increasing age was linked to decreasing Weber fractions (meaning older subjects were more precise in their ANS responses than younger subjects), and the rate of this effect did not differ between the two groups.

#### Relationship Between ANS Performance and Other Tasks

Next, we were interested in the extent to which ANS performance could be predicted by performance on other related tasks. We tested each subject in the following domains: Non-verbal IQ (Raven task), Inhibitory Control (Flanker task), Visual Working Memory, and Attention Network strength. Using the data processing techniques described above, this resulted in the creation of the following scores for each subject: Raven score; Inhibitory Control composite score; Working Memory composite score; and Conflict, Alerting, and Orienting ANT scores (for mean scores on each task, see Table 2). We then predicted Weber fractions from this group of variables, as well as Hearing group and Age. We included interaction terms between Hearing group and each other variable to evaluate whether the pattern of results differed between SHL and SNH.

**TABLE 2:** Mean scores on each task by hearing group.

	**SHL**		**SNH**	
	**Mean**	***SD***	**Mean**	***SD***
Non-verbal IQ (Raven)	48.60	6.12	53.28	6.04
Inhibitory control	158.05	78.92	165.87	80.29
Visual working memory	−0.352	0.79	0.352	0.58
Conflict ANT	131.41	67.63	104.63	55.36
Alerting ANT	29.89	42.77	21.09	39.98
Orienting ANT	−1.47	52.90	8.47	47.38

On the whole, this model explained significant variance in ANS performance, *F*(15, 178) = 3.233, *p* < 0.001, *R*^2^ = 0.148. We found that Age, β = −0.306, *p* = 0.002, Non-verbal IQ, β =−0.238, *p* = 0.029, and Visual Working Memory score, β = −0.201, *p* = 0.046, were each significant predictors when other variables were taken into account. Score on Conflict ANT was marginally significant, β = −0.150, *p* = 0.096, and no other variables were significant, *p*s > 0.116. Increases in each of these variables corresponded to decreases in Weber fractions, indicating that students who were older and had higher Non-verbal IQ, Visual Working Memory capacity, and scores on the Conflict ANT tended to have better ANS acuity. Notably, none of the interactions between group membership and other variables were significant, *p*s > 0.240, indicating that the relationship between ANS and other task performance was similar among SHL and SNH.

Interestingly, hearing group membership was no longer predictive of ANS performance when the other variables were included, β =−0.178, *p* = 0.116. However, due to the decreased power associated with the large number of predictors included in this model, we caution against a strong interpretation of this result.

#### Relationship Between ANS and Mathematics Performance

Finally, we were interested in the degree to which ANS performance could account for variability in formal mathematics scores (*M* = 193.05, *SD* = 62.06), above and beyond that which could be accounted for by other related abilities. We did this by utilizing the suite of predictors tested in the previous section (Hearing group; Age; Raven score; Inhibitory Control composite score; Working Memory composite score; and Conflict, Alerting, and Orienting ANT scores), and used linear regression to determine whether ANS performance predicted Mathematics performance once these variables were taken into account. As in the previous section, the only interactions included in this model were between hearing group membership and each other variable, to determine whether these variables had different explanatory power among SHL compared to SNH.

For our first model, we regressed Mathematics score over the suite of these predictor variables, excluding ANS performance. This model significantly predicted Mathematics ability, *F*(15, 178) = 27.99, *p* < 0.001, *R*^2^ = 0.68 (see Table 3 for standardized coefficients). Of the predictors, only the Attentional Network scores did not significantly explain some variance in Mathematics ability; group membership, Age, Non-verbal IQ, Inhibitory Control, and Visual Working Memory capacity all contributed to explaining Mathematics performance. SNH (*M* = 232.88, *SD* = 41.88) had significantly higher Mathematics scores on average than SHL (*M* = 153.22, *SD* = 52.76). Increasing Age, Non-verbal IQ, and Visual Working Memory capacity corresponded to increases in Mathematics Score. Interestingly, an increase in Inhibitory Control score corresponded to a *decrease* in Mathematics score. No interactions with group membership were significant, *p*s > 0.191, indicating that the influence of each variable on Mathematics score was similar for both groups.

**TABLE 3:** Standardized coefficients from regressions predicting mathematics score.

**Predictor**	**Model 1 β**	**Model 2 β**
Hearing group	0.559***	0.540***
Age	0.149*	0.102.
Non-verbal IQ	0.395***	0.359***
Inhibitory control	−0.125*	−0.122*
Visual working memory	0.146*	0.115.
Conflict ANT	−0.078	−0.101.
Alerting ANT	−0.071	−0.078
Orienting ANT	0.040	0.035
Weber fraction		−0.154**

p < 0.1, *p < 0.05, **p < 0.01, ***p < 0.001. Model 1β, the first model beta-coefficient; Model 2β, the second model beta-coefficient.

**FIGURE 6:**
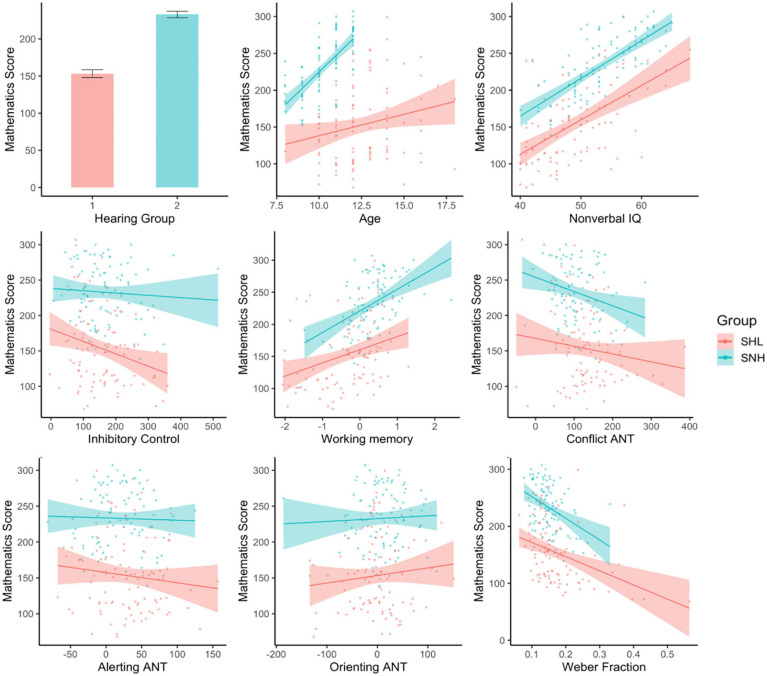
Predicting mathematics score. When other variables were taken into account, only group, Non-verbal IQ, Inhibitory Control, and Weber fraction significantly predicted Mathematics score.

We then compared this model to a second model that included the same predictors and additionally included ANS performance as indexed by Weber fraction. This model also explained a significant amount of variance in Mathematics score, *F*(17, 176) = 26.28, *p* < 0.001, *R*^2^ = 0.69. Weber fractions were significantly predictive of Mathematics score even when other variables were taken into account, *t*(176) = 3.03, *p* = 0.003. Once Weber fraction was added to the model, Age, Visual Working Memory capacity and Conflict ANT score became marginally significant predictors of variance in Mathematics score (likely due to the shared variance between these predictors and ANS performance found in the previous section). Hearing group, Inhibitory Control and Non-verbal IQ remained significant predictors (see Figure 6 for the individual relationship between each predictor and Mathematics score). As in the previous model, there was no interaction between group membership and any of the other predictors, *p*s > 0.111.

We then checked that the second model explained significantly more variance than the first model, to confirm that ANS task performance explained additional variance in our subjects’ Mathematics scores. An ANOVA comparing these two models significantly favored the second model, and therefore the inclusion of ANS task performance, over the first model, *F*(2, 176) = 4.70, *p* = 0.010. ANS ability uniquely explained variance in Mathematics score beyond that which was explained by other predictors, and did so similarly for both SHL and SNH.

## Discussion

To summarize our results, we found that students with hearing loss (SHL) had lower ANS acuity than control subjects (SNH)—even though SHL tended to be a bit older. The magnitude of this effect was decreased when other factors were taken into account (such as Non-verbal IQ and Visual Working Memory capacity), indicating that the difference in ANS performance that we observed may be at least partially due to other factors that tend to vary between these groups, rather than due solely to the imprecision of the ANS representations themselves. All students showed a tendency to perform better on size-confounded than size-controlled trials, consistent with a role for inhibitory control. But, a specific role for reduced inhibitory control to drive especially low ANS acuity in SHL did not bear out. We found that many factors contributed uniquely to performance on the Math test, and most importantly, even when taking these other potential contributing factors into account, the precision of the ANS (Weber fraction) still accounted for significant variance in Math score. Therefore, we conclude that the ANS’s contribution to Math ability in children goes above and beyond that which can be accounted for by other measures such as Inhibitory Control, Working Memory capacity, and Attention Network performance, and, to the extent that we find unique variance between ANS and symbolic math ability above and beyond these factors, these abilities may play only a minor role in modulating the link between the ANS and symbolic math ability.

The present study adds further support for the claim that ANS abilities relate to school math abilities in children, consistent with previous meta-analyses on the topic (Chen and Li, 2014; Schneider et al., 2016). Here, we observed this effect in two large samples of students, controlling for many relevant factors. We also saw that this effect is important both for typically developing children and students with hearing loss (SHL). That we saw accuracy patterns consistent with Weber’s law in our SHL (and only a small difference in Weber fraction between SHL and SNH when controlling for other factors) suggests that the ANS is able to develop somewhat normally in the absence of auditory input. SHL tended to have lower scores on many of the facilities tested in the present studies, which raises the possibility that the ANS deficits we saw are not specific deficits, but rather due to general developmental challenges that arise for deaf children, such as late-onset language exposure or reduced access to early mathematics education (Swanwick et al., 2005; Bull, 2008).

Combining this with the existing result of normal functioning of the ANS in blind participants (Kanjlia et al., 2018) supports the suggestion that the ANS is a domain general cognitive system with representations that abstract away from any particular modal signal. Although size-congruency influenced ANS performance in this sample (and convex hull was not controlled for), given that ANS representations develop in individuals with vastly different sensory experiences, we argue that the content of these shared representations must be something that is preserved across modalities (*see also* Halberda, 2019). That is, if the ANS is able to develop in both blind individuals and SHL, and given that links between the ANS and math ability are observed in both populations, it appears that the ANS abstracts away from particular modal content. Nonetheless, the mechanism underlying congruency effects, and whether they occur at the extraction or response stage, remains a fruitful path for future study.

As with many previous demonstrations, the present results suggest a picture of the ANS as a domain general cognitive system that supports non-symbolic numerical intuitions and relates to symbolic math abilities.

## Data Availability Statement

The raw data supporting the conclusions of this article will be made available by the authors, without undue reservation.

## Ethics Statement

The studies involving human participants were reviewed and approved by the Ethics Committee of Tibet University. Written informed consent to participate in this study was provided by the participants’ legal guardian/next of kin.

## Author Contributions

XB and HM: study concept and design. XB, ES, and TZ: acquisition and analysis or interpretation of data. XB, ES, JH, and HM: drafting of the manuscript. HM and JH: obtained funding. ES, XB, JH, and HM: critical revision of the manuscript for important intellectual content. All authors contributed to the article and approved the submitted version.

## Conflict of Interest

The authors declare that the research was conducted in the absence of any commercial or financial relationships that could be construed as a potential conflict of interest.
